# A Giant Exchange Bias Effect Due to Enhanced Ferromagnetism Using a Mixed Martensitic Phase in Ni_50_Mn_37_Ga_13_ Spun Ribbons

**DOI:** 10.3390/nano13212827

**Published:** 2023-10-25

**Authors:** Fanghua Tian, Qizhong Zhao, Jiale Guo, Sen Kong, Bingjie Liu, Zhiyong Dai, Minxia Fang, Yin Zhang, Chao Zhou, Kaiyan Cao, Sen Yang

**Affiliations:** MOE Key Laboratory for Nonequilibrium Synthesis and Modulation of Condensed Matter, School of Physics, Xi’an Jiaotong University, Xi’an 710049, China; zhaoqizhong@stu.xjtu.edu.cn (Q.Z.);

**Keywords:** Heusler phases, magnetic materials, melt spinning, exchange bias, martensitic structure

## Abstract

The structure of a material is an important factor in determining its physical properties. Here, we adjust the structure of the Ni_50_Mn_37_Ga_13_ spun ribbons by changing the wheel speed to regulate the exchange bias effect of the material. The characterization results of micromorphology and structure show that as the wheel speed increases, the martensite lath decreases from 200 nm to 50 nm, the structure changed from the NM to a NM and 10M mixed martensitic structure containing mainly NM, then changed to NM and 10M where 10M and NM are approaching. Meanwhile, H_E_ first increased and then decreased as the wheel speed increased. The optimum exchange bias effect (H_E_ = 7.2 kOe) occurs when the wheel speed is 25 m∙s^−1^, mainly attributed to the enhanced ferromagnetism caused by part of 10M in NM martensite, which enhanced the exchange coupling of ferromagnetism and antiferromagnetism. This work reveals the structural dependence of exchange bias and provides a way to tune the magnitude of the exchange bias of Heusler alloys.

## 1. Introduction

The structure of materials is crucial to studying them and is the main factor affecting their performance. Different structures and microstructures show different properties [1,2]. The most typical examples are the different physical and chemical properties of materials of the carbon family (such as graphite, diamond graphene, etc.) [3,4,5]. The same phenomenon occurs in magnetic materials [6,7,8,9]. For Fe-Co-V soft magnetic alloys, the occurrence of parts of the γ phase in the α phase will increase the coercive force of the alloy. The brittleness will increase when the ordered α’ phase is generated in the material [6,7]. The structure also plays an important role in studying magnetostrictive materials such as Fe-Ga. Excellent magnetostrictive properties will occur when the m-D0_3_ phase appears in the material [8,9]. Therefore, researching and establishing the relationship between material properties and structures is necessary, which is very important for material design.

The exchange bias (EB) effect refers to the phenomenon of a hysteresis loop deviating from the origin’s center after the magnetic material is cooled with an external magnetic field [10,11,12]. The EB effect has many applications in recording device read heads, electronic spin valves, and magnetoresistive sensors [13,14,15]. This effect exists widely in magnetic systems with magnetic interactions, such as antiferromagnetic/ferromagnetic (AFM/FM) thin films [16,17], FM core/AFM shell nanoparticles [18,19], perovskite systems with phase separation [20,21], and Heulser alloys [22,23]. Ni-Mn base Heusler alloys have received extensive attention and research due to their rich structures (monoclinic, tetragonal, orthorhombic, etc.) and magnetic phase transitions in the martensitic phase [24,25,26]. In Ni_2_Mn_1+x_Z_1−x_ (In, Ga, Sb, etc.) Heusler alloys, the magnetism mainly depends on the distance of Mn atoms, d_Mn–Mn_ < 2.83 Å is AFM, and d_Mn–Mn_ ≥ 2.83 Å is FM; different magnetic states will appear in various structures. In general, the Mn-Mn exchange interaction is FM within the regular Mn sublattices, while it is AFM between the Mn atoms occupying the regular Mn sublattice and Z sublattice. In addition, the tetragonal in the martensitic phase would enhance the AFM, resulting in a significant degree of magnetic frustration, and the orthorhombic distortion would enhance the FM [27,28]. In 2007, Mahmud Khan et al. first discovered the EB effect of 284 Oe in the Heusler alloy [22]. Subsequently, the EB effect of the material was improved by adjusting the alloy atomic ratio [29], component substitution [30], heat treatment process [31], and preparation process [32]. However, the relationship between structure and EB effects has not been studied in depth.

In this work, we prepared Ni_50_Mn_37_Ga_13_ spun ribbons by changing the wheel speed through combined arc melting and using a single-roller melt-spinning apparatus. The relationship between the EB effect and the ribbon structure was studied. The results show that the EB effect has a non-monotonic trend of first increasing and then decreasing with the increase in the wheel speed. An optimal EB effect of 7.2 kOe is obtained when the wheel speed is 25 m∙s^−1^. The structure and microstructure of the material were studied using an X-ray diffractometer (XRD) and transmission electron microscopy (TEM), indicating that the optimal EB effect was caused when the system’s structure was mainly NM in a NM and 10M mixed martensitic structure. This result provides a new method for designing and preparing materials with excellent EB effects.

## 2. Materials and Methods

The Ni_50_Mn_37_Ga_13_ alloy was formulated according to the stoichiometric ratio of 50:37:13 and prepared via arc melting. The ingots were smelted five times, and the purity of the metal elemental raw materials used was 99.9%. Ribbons were produced in a single-roller melt-spinning apparatus with a copper wheel (radius: 12 cm), and the specific processing parameters are shown in Table 1. The as-cast bulk alloys were remelted in a quartz tube with an opening at the bottom via induction melting. After melting, the molten alloy was ejected on the high-speed rotating copper wheel by pressured argon gas. X-ray diffraction (XRD, Bruker D8 Advance X-ray diffractometer, Germany, Cu Kα, λ = 0.15418 nm) was used to investigate the crystalline nature of Ni_50_Mn_37_Ga_13_ ribbons. A field-emission scanning electron microscope (SEM, JEOL JSM-7000F, Japan) with electron energy disperse spectroscopy (EDS) and transmission electron microscopy (TEM, JEOL JEM-2100, Japan) were used to observe the micromorphology and microstructure of the ribbons. The magnetic properties, including magnetic hysteresis (MH) loops and magnetization temperature (MT) curves, are measured using a superconducting quantum interference device (SQUID) magnetometer (Quantum Design, MPMS-XL-5). A differential scanning calorimetry (DSC) at cooling/heating rates of 10 K min^−1^ was used to determine the phase transition temperature.

## 3. Results and Discussion

**Figure 1:**
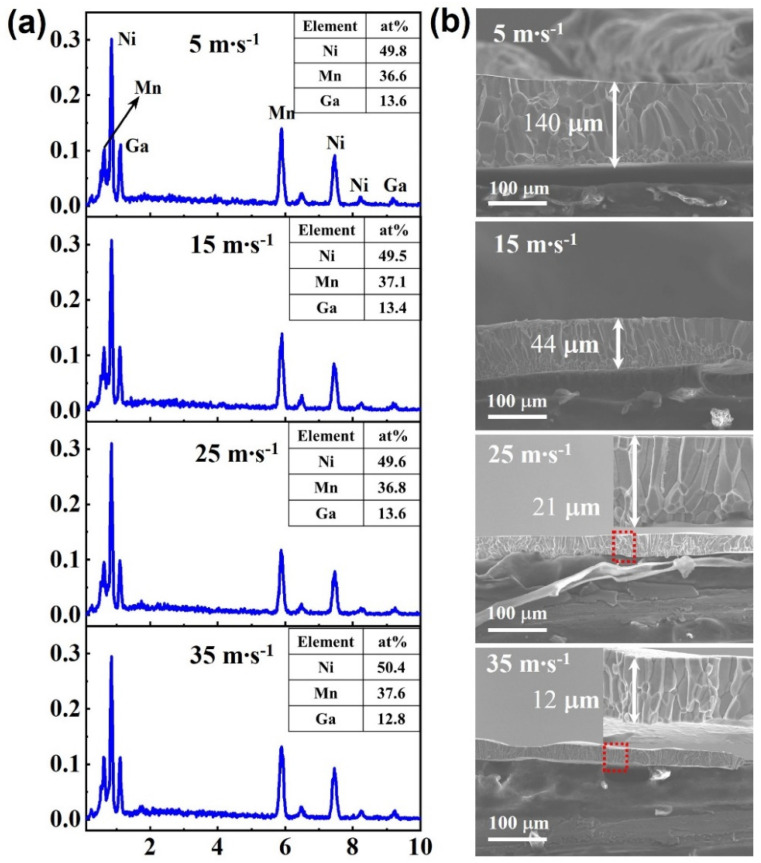
(**a**) EDS analyzed the element ratio of the spun ribbons with different wheel speeds; (**b**) SEM photographs imaged on the fractured cross sections for ribbons with different wheel speeds; the inset is the partially enlarged morphologies of the red box area.

First, the atom ratio of the ribbons was analyzed using EDS. The result is given in Figure 1a, which illustrates analytical spectra and the corresponding alloy composition. It is seen that the composition of the ribbons with different wheel speeds are all very close to the nominal composition and only deviate from an acceptable level. Figure 1b shows the SEM morphologies imaged on the fractured cross sections of the ribbons. When observing the fractured cross sections, the ribbons were placed with the copper wheel contacting face at the bottom and the free face at the top, as shown in Figure 1b. Obvious grain boundaries can be observed on the cross section of all samples, which implies a typical intergranular fracture feature. For the ribbons with relatively low speeds (5 m∙s^−1^ and 15 m∙s^−1^), some equiaxial grains can be observed near the contacting face, while columnar grains appear upwards to the free face. The co-existence of two different types of grains is due to the different cooling rates between the contacting face and the free face due to the relatively thick ribbons. With the increasing wheel speeds, the ribbons become thinner, and the grains also become smaller; only columnar grains can be observed for ribbons with relatively high wheel speeds. The specific thickness of the ribbons is marked in Figure 1b. The thickness of the ribbons when the wheel speed is 5 m∙s^−1^, 15 m∙s^−1^, 25 m∙s^−1^, and 35 m∙s^−1^ is 140 μm, 44 μm, 21 μm, and 12 μm, respectively.

**Figure 2:**
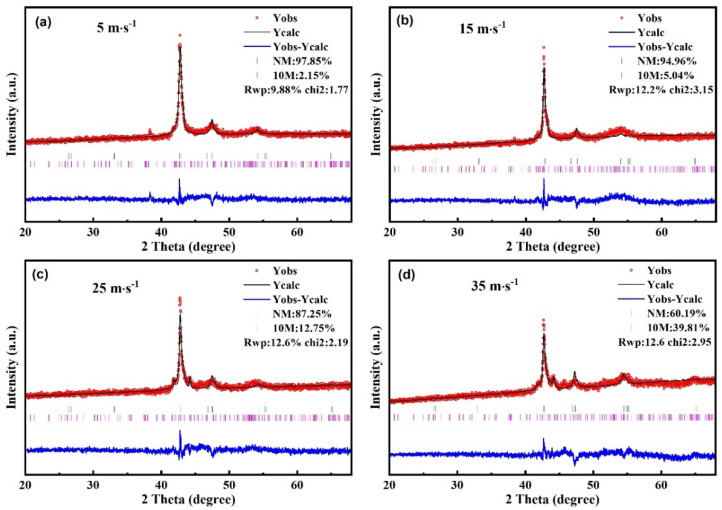
XRD patterns recorded at room temperature for Ni_50_Mn_37_Ga_13_ spun ribbons prepared by different wheel speeds (**a**) 5 m∙s^−1^, (**b**) 15 m∙s^−1^, (**c**) 25 m∙s^−1^, and (**d**) 35 m∙s^−1^.

In order to reveal the structure change of Ni_50_Mn_37_Ga_13_ spun ribbons with wheel speeds of 5~35 m∙s^−1^, X-ray diffraction was carried out at room temperature, as shown in Figure 2. It can be observed that the crystal structure of the two groups of samples with wheel speeds of 5 and 15 m∙s^−1^ is similar to the Ni_50_Mn_37_Ga_13_ bulk alloy (Figure 1a,b) and is mainly the tetragonal NM martensitic phase; the characteristic peaks of the XRD diffraction peaks are (112), (200), (202), and (440) [33]. At the same time, some other weak characteristic peaks also appeared in the diffraction peak pattern at the wheel speed of 15 m∙s^−1^. When the wheel speed increases to 25 m∙s^−1^, in addition to the characteristic diffraction peak of NM, the characteristic peak of the second phase is further enhanced, showing a martensitic structure of 10M, corresponding to the (200), (0010), and (12$\overset{-}{5}$) [34]. When the wheel speed reaches 35 m∙s^−1^, the characteristic peak of 10M martensite is further enhanced. The XRD results were further refined using the Rietveld refinement program of Full Prof. The refinements of NM and 10M are based on the structure from the Inorganic Crystal Structure Database (ICSD); the specific ICSD collection code is 153292 and 158168 for NM and 10M, respectively. Small values of the reliability factors of Rietveld refinement (RWP: weighted profile factor and χ^2^: goodness of fit) indicate a good agreement between experimental and calculated diffraction patterns of samples. The corresponding lattice parameters and volume fractions obtained via the XRD refinements for ribbons with different wheel speeds are summarized in Table 2. The ratios of NM and 10M martensite structures are approximately 98:2, 95:5, 87:13, and 60:40, respectively, when the wheel speed is 5, 15, 25, and 35 m∙s^−1^. The results show that as the wheel speed increases, the dominance of the NM martensite structure changes to NM and 10M, where 10M and NM are approaching. As the structure changes, the distance between Mn atoms changes, thereby changing the magnetic properties of alloys.

**Figure 3:**
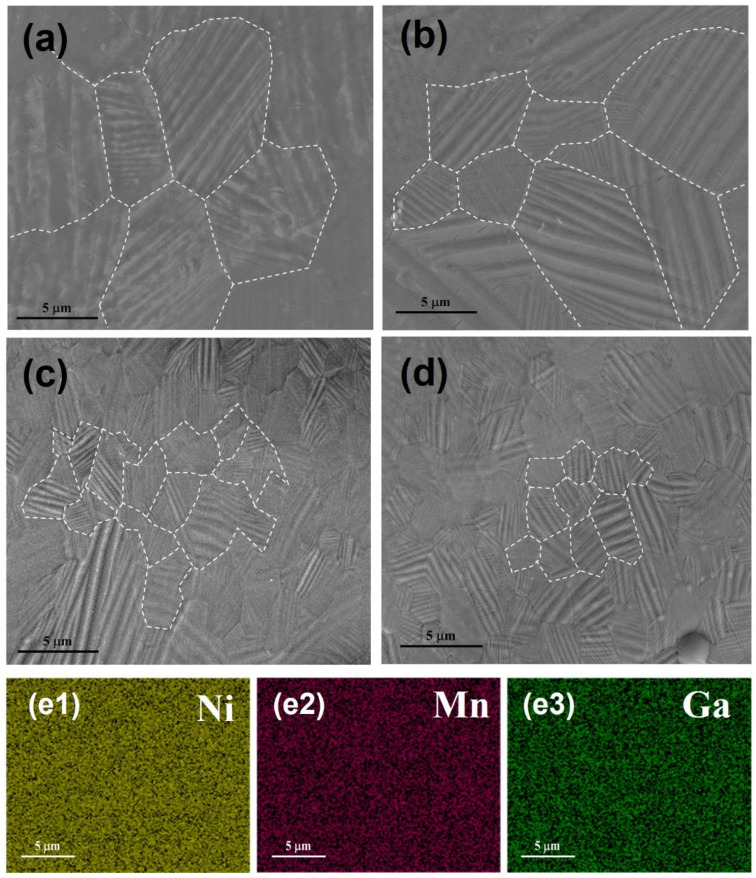
SEM images for Ni_50_Mn_37_Ga_13_ ribbons with different wheel speeds (**a**) 5 m∙s^−1^, (**b**) 15 m∙s^−1^, (**c**) 25 m∙s^−1^, and (**d**) 35 m∙s^−1^. White dashed line is the grain boundaries; (**e1**–**e3**) EDS mapping for SEM image (**c**), the corresponding elemental mapping images of the Ni, Mn, and Ga, respectively.

Figure 3a–d shows the micromorphology of Ni_50_Mn_37_Ga_13_ ribbons. Obvious martensite laths appeared in all the ribbons; however, the directions of adjacent martensite laths are inconsistent, indicating that when martensite encounters martensite of another orientation during the formation and growth process, the growth will stop at the junction between the two. At the same time, it can also be observed that the martensite laths inside the grains are not all parallel, and some of the martensite laths inside the grains are in the shape of spearheads, forming a self-collaborative relationship. With the increase in wheel speed, the lath-like martensite went from coarse to fine. This is because during the single-roller spin quenching and stripping process to prepare the ribbons, the different wheel speeds of the copper rollers lead to differences in the cooling speed of the ribbons, resulting in a slightly different latent heat of crystallization and degree of reaction, so martensite with different crystal orientations or different structures appears, like NM and 10M [35,36]. In addition, an EDS mapping analysis was carried out. The bright spots in different colors correspond to Ni, Mn, and Ga, respectively. The results show that all elements were distributed uniformly throughout the area, indicating no component segregation (Figure 3e1–e3).

**Figure 4:**
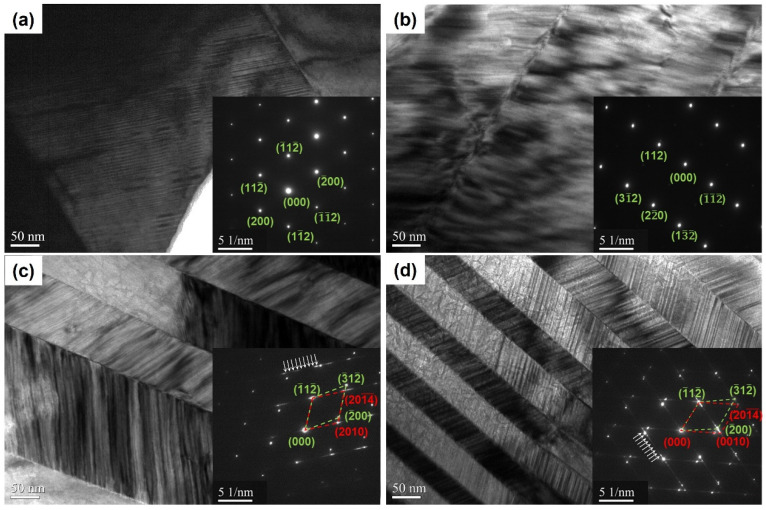
TEM images for Ni_50_Mn_37_Ga_13_ spun ribbons with different wheel speeds (**a**) 5 m∙s^−1^, (**b**) 15 m∙s^−1^, (**c**) 25 m∙s^−1^, and (**d**) 35 m∙s^−1^; inset figure is the corresponding SAED pattern, the arrows indicate the weak spots between the main spots.

To further study the micromorphology and microstructure of the spun ribbons, it was characterized and analyzed using TEM, as shown in Figure 4a–d, and the inset shows that all the ribbons have typical martensitic laths, and the shape and thickness of the laths change in detail with the wheel speed. When the wheel speed is 5 m∙s^−1^, it forms twins in a twin microstructure [37]. The first martensitic twin variant has a width of 500 nm, while the second one has a width of 2 nm. The tetragonal of the corresponding selected area electron diffraction (SAED) pattern was verified as seen in inset Figure 4a; the image is viewed along [021]. The microstructure and diffraction spots of the ribbons with a rotation speed of 15 m∙s^−1^ are similar to those of 5 m∙s^−1^, as shown in Figure 4b. It can be observed from Figure 4c,d that as the wheel speed increases, the martensite laths further reduce, and the width decreases to about 100 nm and 50 nm, respectively. The martensite laths with a wheel speed of 25 m∙s^−1^ show different micromorphology, indicating that the ribbons comprise two types of martensite. As seen in Figure 4c,d, the corresponding SAED represents two sets of overlapping martensitic structures between adjacent martensitic variants. A group of crystal structures is the NM, with nine other diffraction points between the two main diffraction points, which is the 10M crystal structure [38,39]. When the wheel speed is further increased to 35 m∙s^−1^, 10M are approaching NM. With the increase of the wheel speed, the centrifugal force increases dramatically, thus decreasing the thickness of the ribbons and increasing the cooling rate of molten alloys.

Regarding solidification dynamics, ribbons with a high cooling rate would have a high crystallization rate, which could limit the kinetic grain growth and form a small grain [40]. It is the reason for the decrease in grain size with the increasing wheel speed. Meanwhile, from the perspective of solidification thermodynamics, different wheel speeds can also induce different supercooling degrees and form different martensitic phases, such as NM and 10M [41].

**Figure 5:**
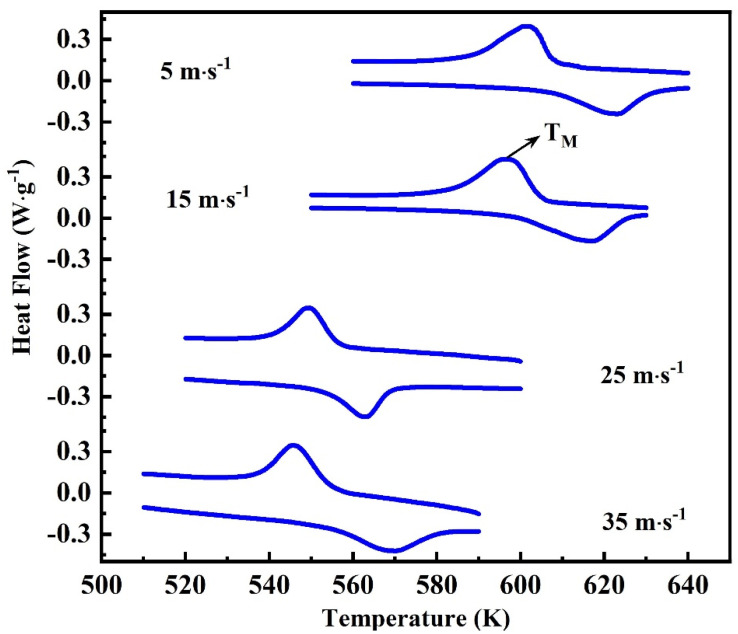
The DSC curves for Ni_50_Mn_37_Ga_13_ ribbons with different wheel speeds 5 m∙s^−1^, 15 m∙s^−1^, 25 m∙s^−1^, and 35 m∙s^−1^.

Figure 5 is the DSC analysis spectrum of Ni_50_Mn_37_Ga_13_ ribbons with different wheel speeds. A pair of obvious endothermic and exothermic peaks appear in all DSC curves, corresponding to the martensitic phase transformation and inverse martensitic phase transformation of the alloy. These two peaks are not in the same position, showing a thermal hysteresis for the transformation. A larger thermal hysteresis indicates that the phase transition is the first-order phase transition. The martensitic transition temperature (T_M_) decreases as the wheel speed increases. The T_M_ for 5 m∙s^−1^, 15 m∙s^−1^, 25 m∙s^−1^, and 35 m∙s^−1^ ribbons are 610 K, 607 K, 560 K, and 556 K, respectively. The decrease in T_M_ is caused by changes in the material structure. 

**Figure 6:**
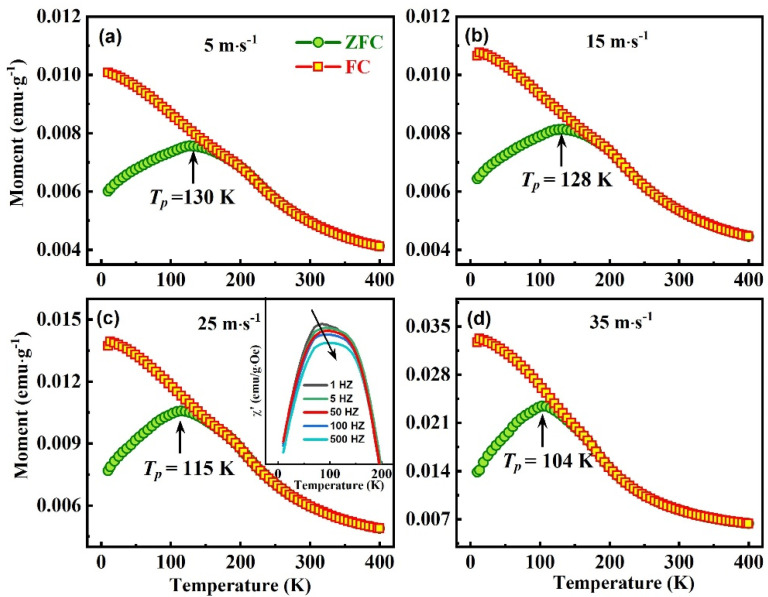
Temperature dependence of magnetization under ZFC/FC processes measured in the presence of a 200 Oe cooling field for Ni_50_Mn_37_Ga_13_ spun ribbons with different wheel speeds (**a**) 5 m∙s^−1^, (**b**) 15 m∙s^−1^, (**c**) 25 m∙s^−1^, and (**d**) 35 m∙s^−1^, respectively. The inset in (**c**) is the AC susceptibility for ribbons with a 25 m∙s^−1^ wheel speed.

Figure 6 shows the MT curves of Ni_50_Mn_37_Ga_13_ spun ribbons under zero-field cooling (ZFC) and field cooling (FC) conditions. The ZFC curve cools the sample from 400 K to 10 K without an external field; then, the temperature is raised to 400 K under an external magnetic field (200 Oe). The change in the magnetization of the sample during the heating process was recorded. The FC curve cools the sample from 400 K to 10 K under 200 Oe and then records the change in magnetization intensity during the heating to 400 K under the same external field. It can be seen in Figure 6a–d that all of the ZFC and FC curves have irreversible magnetization behavior, which could be a typical magnetic feature of spin glass. In order to confirm the occurrence of the spin glass further, an AC susceptibility has been tested, as shown in the inset of Figure 6c. The peak position of the AC susceptibility shifts to higher temperatures with increasing frequency, known as the frequency-diffusion phenomenon, a significant feature of the spin glass [23,29]. The ZFC and FC curves overlap when the temperature exceeds the freezing temperature. When the temperature is lower than the freezing temperature, the ZFC curve and the FC curve are separated, and the magnetization of ZFC is significantly lower than FC. The difference between the ZFC and FC curves increases as the temperature decreases. In addition, the freezing behavior of the magnetic moment begins to occur gradually from above the freezing temperature. As the temperature drops below the freezing temperature, the spins in the ZFC are randomly frozen. In the FC process, under the effect of the cooling field, the spins freeze along the magnetic field direction, distinguished from the random direction of the ZFC process. Thus, the energy barrier in the FC process, which must be overcome during the magnetization process, is smaller than in the ZFC process, which causes a split between the ZFC and FC curves [42]. The peak of the ZFC magnetization curve is the temperature of the spin glass phase transition (*T_p_*). *T_p_* reduced with the wheel speed increase and is 130 K, 128 K, 115 K, and 104 K at 5 m∙s^−1^, 15 m∙s^−1^, 25 m∙s^−1^, and 35 m∙s^−1^, respectively. When the wheel speed increases from 15 m∙s^−1^ to 25 m∙s^−1^ and 25 m∙s^−1^ to 35 m∙s^−1^, two significant Δ*T_p_* mutations occur, which is attributed to changes in the magnetic state caused by structural changes of the NM and 10M martensitic structure. The magnetization intensity of FC increases monotonically with the increase in wheel speed at 10 K, mainly due to the enhanced FM by 10M martensitic structure.

**Figure 7:**
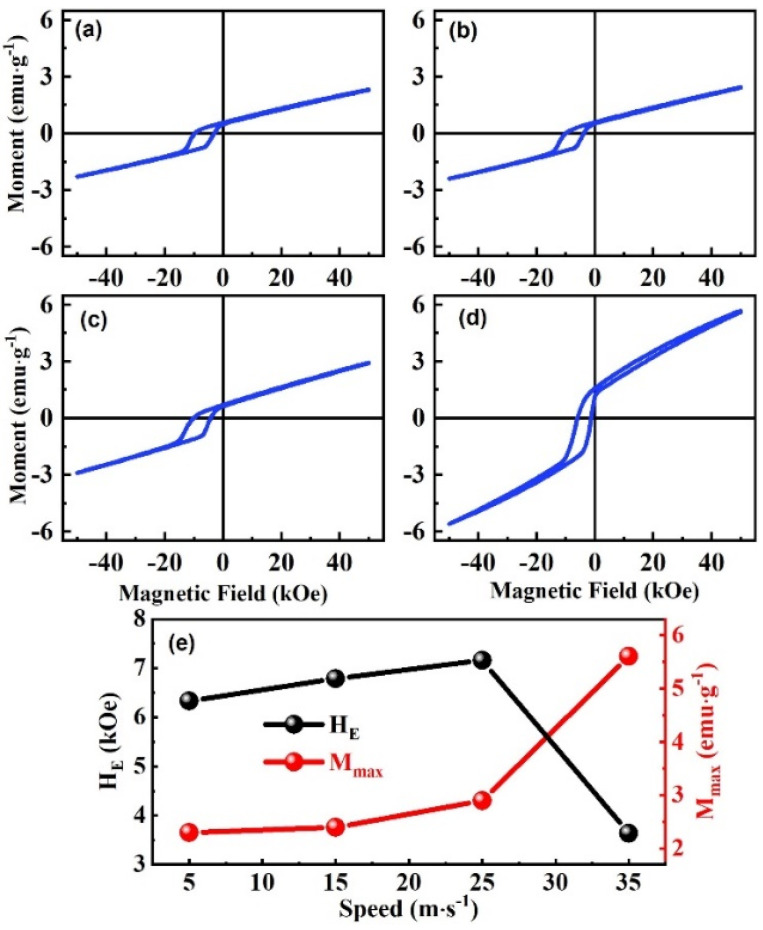
MH loops for Ni_50_Mn_37_Ga_13_ spun ribbons with different wheel speeds (**a**) 5 m∙s^−1^, (**b**) 15 m∙s^−1^, (**c**) 25 m∙s^−1^, and (**d**) 35 m∙s^−1^. (**e**) Dependency of H_E_ and M_max_ with Ni_50_Mn_37_Ga_13_ spun ribbons with different wheel speeds.

As shown in Figure 7, the relationship between the structure and EB was studied via the MH loops of the ribbons at different wheel speeds. All samples were cooled to 10 K under an external cooling field of 20 kOe to test MH loops, and the measurement range was −50 to 50 kOe. In order to avoid the remanence, the sample was first heated to 400 K before the measurement and then cooled down to the measuring temperature. The H_E_ of the EB effect is calculated using H_E_ = −(H_L_ + H_R_)/2. Among them, the H_L_ is the left intersection of the loop with the magnetic field axis, and H_R_ is the right intersection with the magnetic field axis. It can be observed from Figure 7a–d that the shape of MH loops with different wheel speeds are similar, and all have obvious shifts along the magnetic field axis. The MH curves show an obvious hysteresis loop under a low magnetic field, and magnetization increases linearly in the region of the high magnetic field. Magnetization loops are attributed to the co-existence of the FM and AFM phases, and the high field linear part is from the contribution of AFM phases [43]. The interaction of the FM and AFM phases causes the EB effect. As shown in Figure 7e, H_E_ shows a non-monotonic trend of first increasing and then decreasing as the wheel speed increases. When the wheel speed is 5 m∙s^−1^, the H_E_ is 6.3 kOe, and the optimal EB effect with H_E_ = 7.2 kOe appears at the wheel speed 25 m∙s^−1^. Then, as the wheel speed increases further, the H_E_ is reduced to 3.6 kOe. This was caused by changing the martensitic structure from the NM to the mixed martensitic structure of NM and 10M, then changing to NM and 10M, where 10M and NM are approaching. The optimal EB effect occurs in the ribbon that is mainly NM and contains part of 10M martensite. This is attributed to the enhancement of FM by a small amount of 10M martensitic structure, resulting in an enhanced exchange coupling between FM and AFM. The maximum magnetization (M_max_) is the magnetization measured at the maximum measuring field (50 kOe) and can express the difficulty of the magnetization. Moreover, the M_max_ of the spun ribbons increases from 2.3 emu∙g^−1^ to 2.4 emu∙g^−1^; when the wheel speed is 5 and 15 m∙s^−1^, M_max_ does not change much. At this time, the martensitic structure is mainly NM. The magnetization significantly varies from 2.4 to 3.0 emu∙g^−1^ when the wheel speed increases to 25 m∙s^−1^. This is due to the further formation of more 10M. When the wheel speed is further increased to 35 m∙s^−1^, the M_max_ undergoes a mutation and increases to 5.6 emu∙g^−1^, caused by a mixed structure of 10M approaching NM.

**Figure 8:**
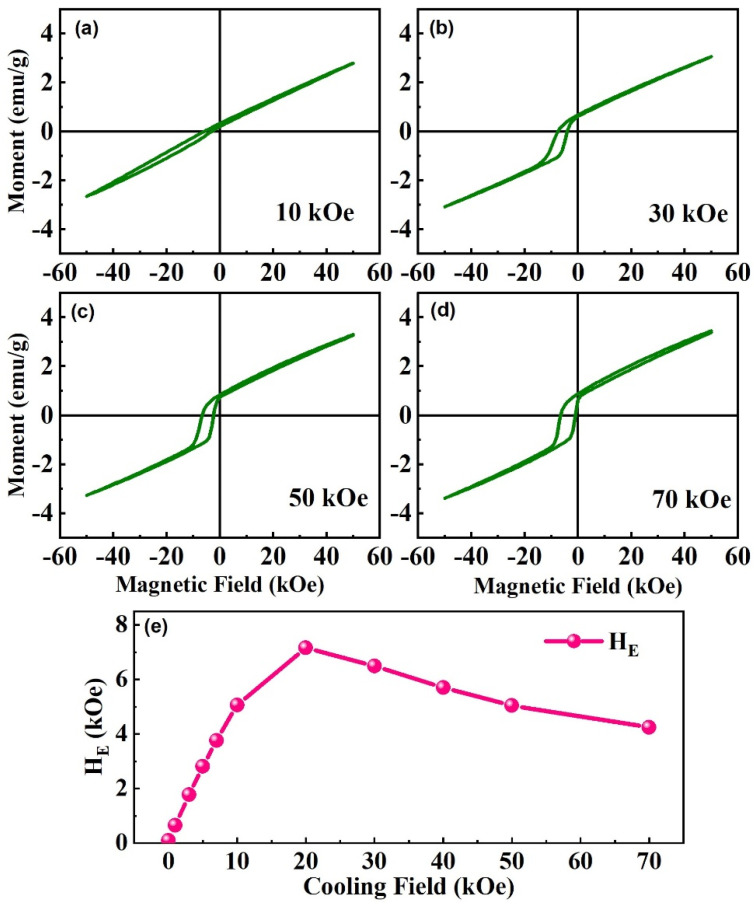
MH loops for Ni_50_Mn_37_Ga_13_ spun ribbons for 25 m∙s^−1^ after cooling to 10 K with different cooling fields (**a**) 10 kOe, (**b**) 30 kOe, (**c**) 50 kOe, and (**d**) 70 kOe. (**e**) Dependency of H_E_ with Ni_50_Mn_37_Ga_13_ spun ribbons with different cooling fields.

As shown in Figure 8, the center of the MH loop also shifts toward negative magnetic moments caused by spin glass behavior and the competition between FM and AFM [42]. This shift is strongly dependent on the cooling field, as shown in Figure 8a–d. The centers of the MH curves shift to a negative moment, weaken with the increasing cooling field, and confirm that this shift is a characteristic of the system itself, not caused by the measurement. Furthermore, the EB effect shows a tendency to first increase and then decrease with the increasing cooling field and obtain a peak value under a 20 kOe cooling field (Figure 8e). With the expansion of the cooling field, the FM cluster grows bigger, thus increasing the FM interaction and changing the competition between the FM and AFM [29]; the symmetry along the moment axis and EB effect changes correspondingly. Finally, when the cooling field is increased to 70 kOe, the EB still remains, but the shift of MH loops along the moment axis almost disappears, as shown in (Figure 8d).

## 4. Conclusions

In summary, we prepared the Ni_50_Mn_37_Ga_13_ spun ribbons by changing the wheel speed (5~35 m∙s^−1^) through a single-roller melt-spinning apparatus. The martensitic structure changed from NM to NM and 10M, where NM becomes dominant, then changed to NM and 10M, where 10M and NM are approaching. The optimal EB effect with H_E_ = 7.2 kOe appears when the wheel speed is 25 m∙s^−1^, which is attributed to the structure of the spun ribbon being mainly NM and containing a certain amount of 10M martensite; the emergence of the 10M structure enhances the FM of the system, thereby enhancing the exchange coupling between FM and AFM. Our work concluded the relationship between structure and EB effects, providing an effective way to design functional materials with excellent EB effects.

## Figures and Tables

**Table 1:** The specific processing parameters used in the melt-spinning method.

	Liner Speed (m∙s^−1^)	Rotate Speed (r∙min^−1^)	Induction Current (A)	Pressure (Mpa)
1	5	400	30	0.13
2	15	1200	30	0.13
3	25	2000	30	0.13
4	35	2800	30	0.13

**Table 2:** The corresponding lattice parameters and volume fractions using XRD refinements for ribbons with different wheel speeds.

Wheel Speeds	Martensitic Structure	a (Å)	b (Å)	c (Å)	α (°)	β (°)	γ (°)	Fraction (%)
5 m∙s^−1^	NM	3.82890	3.82890	6.76937	90	90	90	97.85
	10M	4.31779	5.74124	21.1100	90	94.06709	90	2.15
15 m∙s^−1^	NM	3.82549	3.82549	6.79031	90	90	90	94.96
	10M	4.32860	5.73045	21.21901	90	93.82677	90	5.04
25 m∙s^−1^	NM	3.83053	3.83053	6.75827	90	90	90	87.25
	10M	4.32496	5.74897	21.17277	90	93.83138	90	12.75
35 m∙s^−1^	NM	3.84313	3.84313	6.733910	90	90	90	60.19
	10M	4.33453	5.78678	20.76578	90	93.7393	90	39.81

## Data Availability

The data presented in this study are available upon request from the corresponding author.
